# Neutrophil-to-lymphocyte ratio as a gene-specific biomarker of JAK2-driven inflammation across CHIP and myeloproliferative neoplasms

**DOI:** 10.3389/fonc.2026.1926319

**Published:** 2026-08-11

**Authors:** Tiziano Barbui

**Affiliations:** FROM - Research Foundation Bergamo Hospital - Ente del Terzo Settore (ETS), Bergamo, Italy

**Keywords:** CHIP, JAK2V617F, molecular monitoring, myeloproliferative neoplasm, neutrophil-to-lymphocyte ratio, ropeginterferon

## Abstract

The neutrophil-to-lymphocyte ratio (NLR) has emerged as a mechanistically informative biomarker in clonal hematopoiesis of indeterminate potential (CHIP) and myeloproliferative neoplasms (MPNs), although its clinical utility is strongly mutation dependent. NLR is markedly elevated in *JAK2V617F*-mutated CHIP and MPNs, reflecting neutrophil-driven myeloproliferation, whereas it remains largely unchanged in *TET2*, *DNMT3A*, and *ASXL1* mutations despite substantial IL-1β- and IL-6-mediated inflammation. This apparent paradox highlights distinct inflammatory programs generated by different driver mutations, with JAK2 promoting neutrophilia and epigenetic mutations primarily activating macrophage-mediated innate immunity. In polycythemia vera, elevated NLR independently predicts thrombosis, myelofibrotic progression, and mortality. Similar prognostic value has been reported in essential thrombocythemia and myelofibrosis, particularly among patients with *JAK2* mutations. Beyond risk stratification, NLR also functions as a pharmacodynamic biomarker. During treatment with ropeginterferon alfa-2b, reductions in NLR parallel decreases in *JAK2V617F* allele burden, whereas persistent NLR elevation during hydroxyurea therapy identifies patients at higher risk of thrombosis and adverse outcomes. Conversely, the CANTOS trial demonstrates that patients with *TET2*-mutated CHIP derived marked cardiovascular benefit from IL-1β inhibition despite NLR providing limited biological information, underscoring the need for complementary biomarkers in non-JAK2 CHIP. This review integrates mechanistic, clinical, and therapeutic evidence supporting mutation-specific inflammatory biomarkers and proposes NLR-guided strategies for risk assessment, treatment monitoring, and precision management in MPNs and CHIP.

## 1 Introduction

Clonal hematopoiesis of indeterminate potential (CHIP) and myeloproliferative neoplasms (MPNs) represent a continuum of clonal blood disorders with distinct thrombotic, hemorrhagic, and malignant transformation risks. CHIP, defined by somatic mutations in hematopoietic genes (*JAK2, TET2, DNMT3A, ASXL1*) detected at ≥2% variant allele frequency (VAF) without peripheral blood count modifications or splenomegaly, affects ~10% of older adults and increases cardiovascular and hematologic malignancy risk 1.3- to 2.0-fold (1).

MPNs, comprising polycythemia vera (PV), essential thrombocythemia (ET), and primary myelofibrosis (PMF), are characterized by constitutive JAK-STAT signaling, excessive myeloid proliferation, and thrombotic complications responsible for 25–40% of MPN mortality (2).

The neutrophil-to-lymphocyte ratio (NLR), calculated from absolute neutrophil and lymphocyte counts on routine complete blood count, has historically served as a non-specific inflammatory marker in oncology and cardiovascular disease. NLR reflects the balance between innate inflammatory activation and adaptive immune competence. In MPNs, neutrophils display marked functional heterogeneity and circulate in a chronically activated state, producing inflammatory mediators and suppressing T-cell and natural killer cell cytotoxicity (3).

This dual role promotes both chronic inflammation and impaired immune surveillance, potentially facilitating clonal expansion, disease progression, and immune escape. Although neutrophilia has traditionally been viewed as a surrogate of inflammation, this Perspective argues that the prognostic value of NLR lies in the imbalance between innate and adaptive immunity rather than neutrophilia alone. NLR should therefore be considered a composite biomarker reflecting the inflammatory consequences of *JAK2*-driven myelopoiesis.

Consequently, NLR provides information on inflammatory activity and immune dysfunction beyond absolute neutrophil or lymphocyte counts, making it a clinically relevant biomarker of disease biology and prognosis. Compared with total white blood cell count, NLR is a stronger predictor of cardiovascular events (4) and mortality (5).

Of particular interest, recent evidence indicates that the clinical utility of NLR in CHIP and MPNs is fundamentally mutation dependent and mechanistically informative. *JAK2V617F*-driven clones produce marked neutrophilia through constitutive granulopoiesis, resulting in substantial NLR elevation. In contrast, epigenetic mutations such as *TET2, DNMT3A*, and *ASXL1* generally do not produce comparable increases in NLR despite eliciting robust, clinically relevant inflammation. Rather than promoting neutrophil expansion, these mutations predominantly activate macrophage-mediated inflammatory pathways characterized by enhanced IL-1β and IL-6 signaling. This mutation-specific divergence explains why NLR is a powerful biomarker in JAK2-driven disease but has more limited value in non-JAK2 CHIP, where complementary inflammatory biomarkers are required. Taken together, these observations support the hypothesis that NLR may function as a biomarker preferentially associated with JAK2-driven inflammation.

In clinical practice, NLR serves four complementary roles (6): (1) risk stratification in asymptomatic CHIP and MPN, identifying high-risk thrombotic phenotypes (2) pharmacodynamic monitoring of JAK inhibitor and interferon response providing early signals of molecular control (5); (3) adverse event prediction, particularly immune-related complications (7); and (4) integration into multi-parameter algorithms that combine NLR with gene-specific mutations, mutational burden, and alternative inflammatory markers (IL-1β/IL-6, hsCRP) to refine personalized risk estimates (8).

This review synthesizes recent mechanistic, clinical, and therapeutic advances to establish NLR as a gene-specific, actionable biomarker in CHIP and MPNs, while highlighting the critical distinction between quantitative neutrophilia (JAK2-driven) and qualitative macrophage-mediated inflammation (epigenetic mutations driven) that current NLR alone cannot capture.

## 2 NLR as a gene-specific biomarker: distinct inflammatory programs in JAK2- and non-JAK2–mutated disease

### 2.1 In CHIP, NLR levels are heterogeneous

Gumuser et al. analyzed 13,129 individuals (median age 63 years), identifying 665 (5.1%) subjects with CHIP (9). *DNMT3A* CHIP was detected in 337 participants (2.6% of overall cohort), *TET2* CHIP in 92 (0.7%), *ASXLl* CHIP in 141 (1.1%), *JAK2* CHIP in 9 (0.1%), *PPM1D/TP53* CHIP in 41 (0.3%), and *SF3B1/SRSF2/U2AF1* CHIP in 30 (0.2%). *DNMT3A, TET2*, and *ASXL1* CHIP collectively constituted 83.9% (n = 558 of 665) of CHIP cases. Overall, CHIP-positive individuals exhibited only a modest increase in NLR compared with CHIP-negative controls. Median high-sensitivity C-reactive protein (hsCRP) was slightly higher in individuals with CHIP than in those without CHIP, although the proportion of patients with hsCRP ≥2 mg/L did not differ significantly. Likewise, NLR was only modestly elevated in CHIP carriers, suggesting that NLR is unlikely to represent a universal biomarker of CHIP-associated inflammation.

However, mutation-specific analysis revealed that JAK2-mutated CHIP represented a distinct inflammatory phenotype, with a median NLR of 4.5 (IQR 3.0–6.2), significantly higher than both CHIP-negative individuals and carriers of non-JAK2 CHIP mutations. These differences remained significant after exclusion of participants with prevalent cancer and after adjustment for age and sex. Although both hs-CRP and NLR were independently associated with adverse cardiovascular outcomes, adjustment for these inflammatory biomarkers did not attenuate the associations between CHIP genotypes and clinical outcomes (9).

Therefore, this gene-dependent NLR heterogeneity reflects fundamentally distinct biological mechanisms. *JAK2V617F* activates canonical JAK-STAT signaling in myeloid progenitors, driving constitutive granulopoiesis through STAT5-mediated upregulation of G-CSF signaling and myeloid lineage commitment.

This notion was confirmed in a recent population-based cohort of 19,832 individuals screened for *JAK2V617F* and CALR mutations by Larsen et al. (10) These authors demonstrated that the biological consequences of clonal hematopoiesis are gene-specific rather than simply clone-size dependent. Even very small *JAK2V617F* clones were associated with a significantly increased risk of cancer, whereas *CALR* mutations showed no significant association. Importantly, only JAK2-mutated CH exhibited a measurable inflammatory response, as reflected by NLR. While an elevated NLR had little effect in individuals without CH, it identified a substantially higher cancer risk in those carrying JAK2-mutated clones. These findings suggest that even low-burden *JAK2* mutations are sufficient to trigger a biologically detectable innate immune response, whereas *CALR*-mutated clones appear to induce little or no systemic inflammatory activation. Consequently, NLR emerges as a simple surrogate biomarker of *JAK2*-driven inflammation.

Mechanistic studies showed that neutrophil-restricted expression of *JAK2V617F* is sufficient to drive a pro-inflammatory phenotype in MPNs, whereas *CALRdel52* is not (11). In *JAK2V617F* mice, plasma levels of IL-1α, IL-12, and M-CSF were significantly increased, with trends toward higher IL-1β, TNF-α, and IL-17. *JAK2V617F* neutrophils also exhibited increased VLA-4 integrin activation, enhanced endothelial adhesion, and impaired migration. These findings provide mechanistic evidence that mutant neutrophils actively sustain inflammation and may contribute to the higher thrombotic risk associated with *JAK2V617F* compared with *CALR*-mutated myeloproliferative neoplasms.

Khatib-Massalha et al. provided preclinical *JAK2V617F* mouse models, with validation in human MPN samples, to show that mutant neutrophils evade macrophage-mediated clearance through upregulation of the immune checkpoint molecule CD24 (12). Defective efferocytosis led to the accumulation of senescent neutrophils, sustained inflammation, and accelerated bone marrow fibrosis. Pharmacologic and genetic blockade of the CD24–Siglec axis restored neutrophil clearance and reduced fibrosis in mice. These findings identify defective neutrophil clearance as a potential therapeutic target, although no clinical intervention was performed in patients.

By contrast, *TET2, DNMT3A*, and *ASXL1* mutations operate through epigenetic reprogramming rather than growth factor pathway activation. These mutations drive clonal expansion through altered histone acetylation and DNA methylation states that enhance hematopoietic stem cell self-renewal and quiescence, but do not inherently increase myeloid differentiation. Instead, they reprogram macrophage phenotypes toward pro-inflammatory (M1) states characterized by elevated IL-1β, IL-6, TNF-α, and reactive oxygen species production, while simultaneously suppressing anti-inflammatory (M2) polarization (13). This qualitative shift in macrophage behavior, not quantitative neutrophil expansion, drives systemic inflammation. Consequently, *TET2-, DNMT3A-*, and *ASXL1*-mutated CHIP patients exhibit elevated circulating cytokines (IL-1β, IL-6) without neutrophilia, explaining why NLR fails to capture their inflammatory burden and why alternative biomarkers (plasma IL-1β, hsCRP) may better reflect their cardiovascular risk (14).

Accordingly, NLR may be less informative in epigenetic CHIP than in *JAK2*-mutated CHIP, but this biologically plausible hypothesis, supported by mechanistic studies, warrants prospective validation (9).

## 3 NLR in MPN risk stratification: static and dynamics application

Accumulating evidence indicates that the clinical value of NLR differs substantially across MPN subtypes.

In a nationwide Danish population-based study (15), NLR independently predicted all-cause mortality in MPN patients, with a markedly stronger prognostic impact than in the general population. Although increasing NLR was associated with progressively worse survival across the MPN spectrum, its discriminatory value was greatest in PV and ET, whereas in MF its incremental prognostic contribution was less pronounced. These observations support the concept that NLR is most informative during the earlier, JAK2-driven phases of MPN evolution, when neutrophilic inflammation remains a major determinant of thrombotic and clinical outcomes. Whether this paradigm also applies to CALR-mutated disease remains largely unresolved.

The evidence reviewed so far indicates that NLR is not merely a baseline prognostic biomarker but a dynamic readout of *JAK2*-driven disease biology. This distinction has important therapeutic implications.

### 3.1 Baseline NLR in polycythemia vera

Polycythemia vera provides the strongest clinical evidence supporting NLR as a biomarker of *JAK2*-driven inflammation (4, 5, 16). In PV, the ECLAP (European Collaboration on Low-dose Aspirin in Polycythemia Vera) prospective cohort of 1,508 patients established NLR ≥5.0 as an independent predictor of venous thrombosis with hazard ratio 2.13 (95% CI 1.56–2.91), independent of traditional risk factors (age, JAK2 burden, hematocrit control (4). The prognostic superiority of NLR versus leucocyte counts in PV extends to overall survival and leukemic transformation. Patients with baseline NLR ≥4.5 experienced shorter median survival and higher AML transformation rates compared to NLR <3.5 cohorts, suggesting that elevated NLR captures both thrombotic and malignant transformation risks (5).

As a dynamic inflammatory biomarker, NLR may be influenced by intercurrent infections, corticosteroid therapy, smoking, autoimmune diseases, and other inflammatory conditions. When appropriate, we accounted for this potential variability using a joint modeling approach, in order to confirm that persistently elevated NLR remained independently associated with thrombosis and mortality, supporting its role as a robust marker of sustained disease-related inflammation.

### 3.2 Baseline NLR in essential thrombocythemia

Although NLR has emerged as a promising inflammatory biomarker in ET, its predictive value has not been adequately validated within molecularly defined subgroups. In particular, whether NLR retains prognostic significance in CALR-mutated disease remains largely unresolved. Available studies report driver mutation status and confirm the lower thrombotic risk of CALR-mutated ET, but they do not provide evidence that NLR performs differently in *JAK2-* and *CALR*-mutated patients in whom CALR-mutated disease may follow a different inflammatory and vascular biology (17).

Future studies should determine whether mutation-specific inflammatory biomarkers differ across these molecular subtypes.

### 3.3 Baseline NLR in primary myelofibrosis

The predictive value of NLR for thrombotic risk was determined in a multicenter cohort of 225 patients with prefibrotic myelofibrosis (prePMF) (18). After a median follow-up of 5.9 years, 37 thrombotic events occurred in 31 patients (2.5 events/100 patients/year; 18 arterial, 19 venous). The patients with NLR ≥ 6 showed a shorter venous thrombosis-free survival (*p* = 0.003) but no significant association with total and arterial thrombotic events was found. In primary myelofibrosis (PMF), NLR ≥2.0 predicts inferior overall survival (HR 2.76) and identifies patients unlikely to respond to ruxolitinib therapy (JAK1/2 inhibitor).

In overt-PMF, Laganà et al. showed that baseline NLR independently predicted ruxolitinib response, with NLR <2.5 patients experiencing greater symptom improvement and superior event-free survival compared to NLR≥2.5 cohorts (19). Paradoxically, very high baseline NLR (≥5.0) in PMF appears associated with less aggressive clonal disease (lower VAF, slower progression), possibly reflecting compensatory immune activation in high-burden disease.

This NLR-outcome relationship in MF highlights the importance of integrating NLR with additional molecular parameters (*ASXL1*, *SRSF2* mutations, karyotype) for comprehensive risk stratification. An important unresolved issue is whether the prognostic value of NLR extends to *CALR*-mutated myelofibrosis. Prospective studies stratified by driver mutation are therefore needed to determine whether NLR retains prognostic and predictive value across distinct molecular subtypes.

Taken together, these studies in MPNs indicate that NLR should not be regarded as a universal inflammatory marker across all MPNs. Rather, its clinical utility appears greatest in *JAK2*-driven disease, where neutrophilia is an integral component of disease biology. Conversely, the evidence supporting NLR in CALR-mutated ET and MF remains limited, reinforcing the hypothesis that the prognostic significance of NLR is determined more by the underlying molecular driver than by the MPN phenotype itself.

## 4 Dynamic NLR as pharmacodynamic biomarker

Unlike fixed risk factors, NLR can be measured serially, allowing clinicians to monitor whether treatment effectively suppresses the inflammatory program associated with the malignant clone. Consequently, changes in NLR may provide an early pharmacodynamic signal of biological response, complementing molecular endpoints such as JAK2V617F allele burden.

To investigate the clinical significance of NLR dynamics in PV, we analyzed three complementary studies representing distinct therapeutic settings and stages of disease (5). First, we examined a propensity score-matched cohort from the ECLAP study, comparing 207 patients treated with hydroxyurea (HU) with 207 patients managed with phlebotomy alone. This analysis assessed whether conventional HU cytoreductive therapy modifies longitudinal NLR and whether persistent NLR predicts thrombosis and survival independently of leukocytosis.

Second, we evaluated the randomized Low-PV phase II trial, which compared ropeginterferon alfa-2b (Ropeg) with phlebotomy alone in 126 low-risk patients. This study investigated whether early interferon therapy produces greater reductions in NLR than standard treatment and whether these changes parallel clinical and molecular responses.

Finally, we analyzed data from the PROUD-PV/CONTINUATION-PV trial, directly comparing Ropeg with HU. These studies explored the relationship between longitudinal NLR dynamics, reduction of *JAK2V617F* variant allele frequency (VAF), and long-term clinical outcomes, providing insight into the biological mechanisms underlying disease modification.

### 4.1 Hydroxyurea failed to normalize NLR: evidence from the ECLAP propensity score analysis

**Figure 1:**
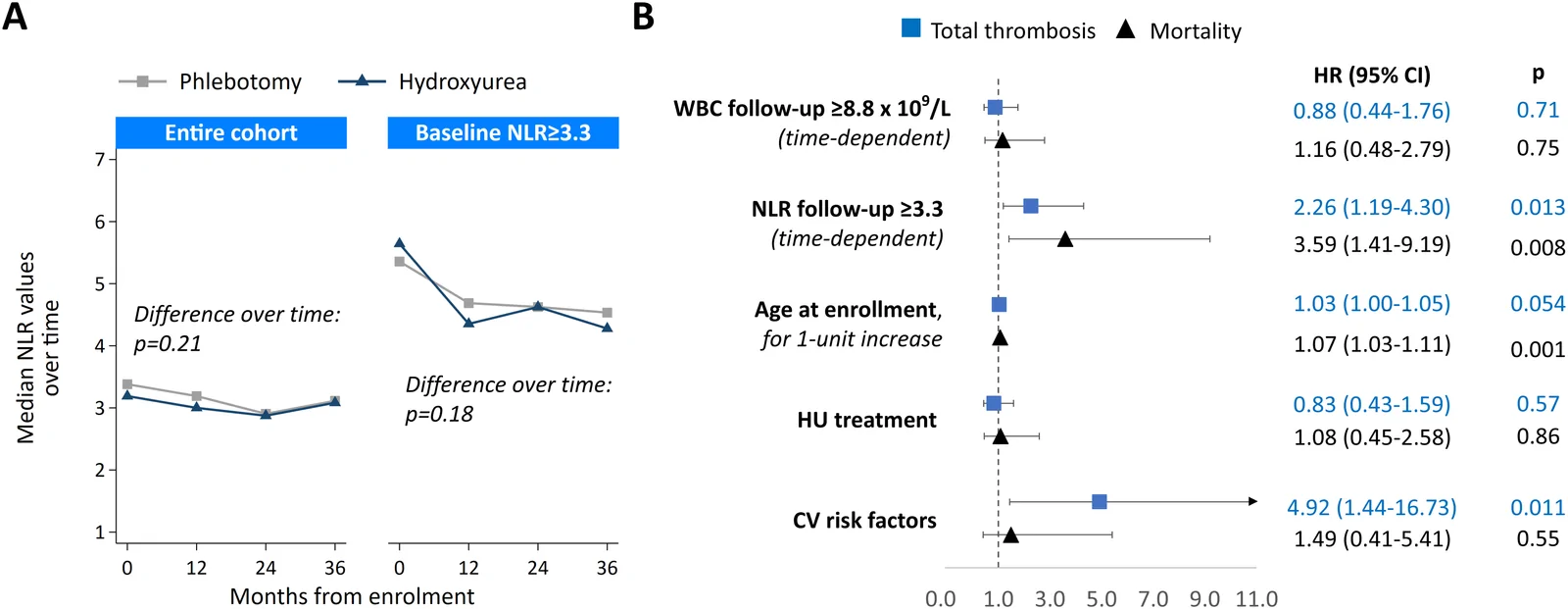
Impact of HU on NLR dynamics and prognostic relevance for thrombosis and mortality. **(A)** Longitudinal analysis of propensity score-matched patients showed that hydroxyurea did not significantly reduce NLR compared with phlebotomy alone over 36 months, either in the overall cohort or among patients with baseline NLR ≥3.3. **(B)** reports the time-dependent multivariable Cox analysis evaluating predictors of total thrombosis and overall mortality during follow-up.

As shown in **Figure 1A**, HU did not produce a sustained reduction in median NLR over 36 months compared with phlebotomy alone, either in the overall cohort or in the subgroup of patients with elevated baseline NLR (≥3.3). This pattern was also confirmed by using joint modeling approach. Thus, despite effective control of myeloproliferation, HU failed to normalize the inflammatory phenotype reflected by NLR.

Persistent elevation of NLR during follow-up (NLR ≥3.3) independently predicted both total thrombosis (HR 2.01, 95% CI 1.04–3.88; *P* = 0.038) and overall mortality (HR 4.49, 95% CI 1.64–12.31; *P* = 0.003). By contrast, time-dependent leucocytosis (WBC ≥8.8 × 10^9^/L) was not independently associated with either thrombosis or mortality after adjustment for NLR and other covariates (**Figure 1B**).

This finding has important biological implications. Once NLR was introduced into the multivariable model, leucocytosis lost its prognostic significance, indicating that NLR captures the clinically relevant information conveyed by leukocyte count while simultaneously integrating the adaptive component of the inflammatory response. In other words, NLR outperformed leucocytosis as a predictor of long-term vascular complications and survival. Therefore, serial NLR assessment identifies residual biological risk that is not captured by conventional hematologic response criteria and represents a more comprehensive biomarker of treatment efficacy.

**Figure 2:**
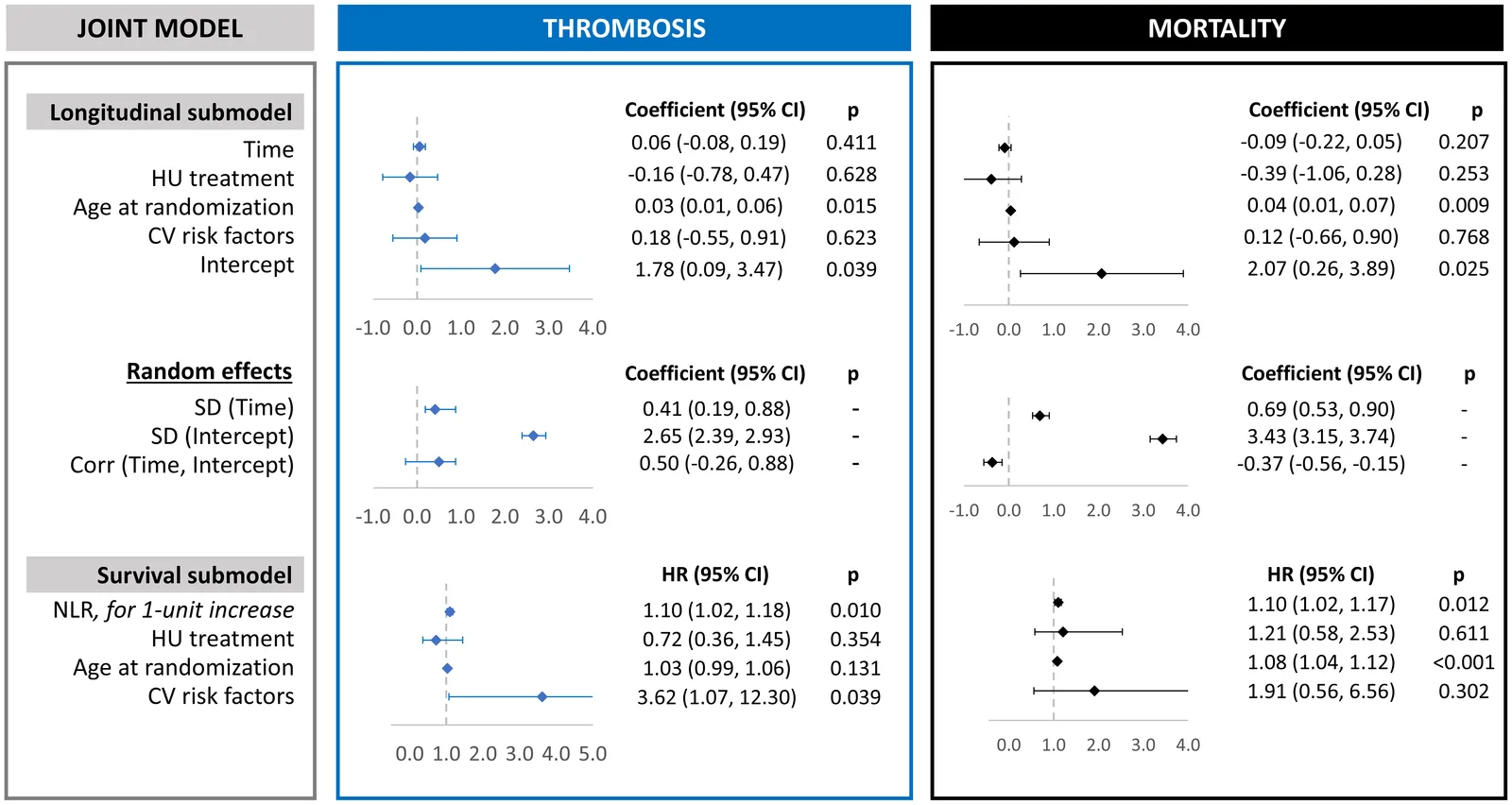
Sensitivity analysis of the association between longitudinal NLR and risk of thrombosis and mortality: results from joint models.

Because NLR is a dynamic inflammatory biomarker, individual measurements may fluctuate over time owing to transient inflammatory conditions, including intercurrent infections, potentially obscuring the relationship between chronic inflammation and clinical outcomes. To address this limitation, we performed a joint modeling analysis, which simultaneously evaluates longitudinal NLR trajectories and time-to-event outcomes (**Figure 2**) (5).

Unlike conventional Cox models, joint models estimate each patient’s underlying NLR trajectory while accounting for measurement variability and informative dropout after clinical events. This approach demonstrated that persistent elevation of NLR was independently associated with a significantly higher risk of both thrombosis and mortality, confirming NLR as a robust marker of sustained disease-related inflammation.

### 4.2 NLR dynamics under ropeg and ruxolitinib

Ropeg appears to be most effective in PV patients with a stronger inflammatory profile, reinforcing the link between inflammation and clonal expansion (5, 16).

In the Low-PV study, patients with baseline NLR≥3.5 had a substantially higher JAK2V617F variant allele frequency than those with lower NLR, together with a more proliferative phenotype. Ropeg was more effective than phlebotomy alone at reducing NLR, primarily by reducing neutrophils while preserving lymphocytopenia. This decrease was directly linked to JAK2V617F suppression and the achievement of the primary endpoint.

**Figure 3:**
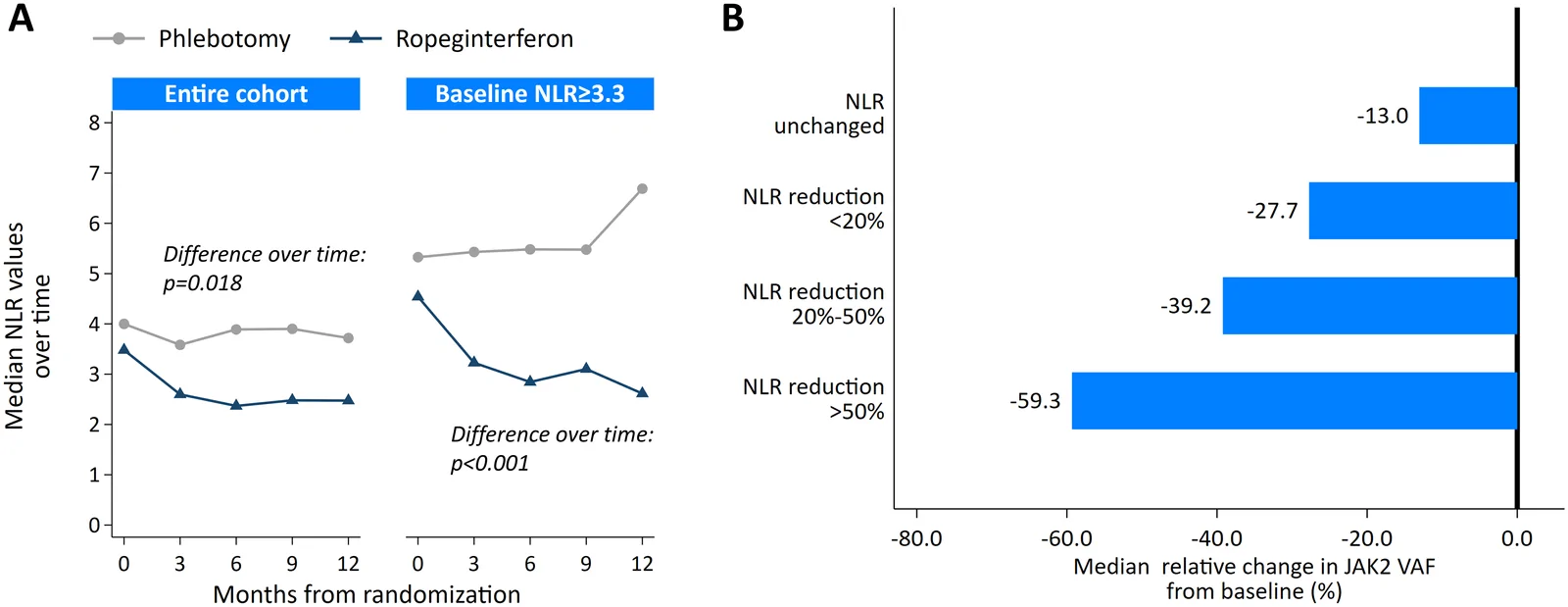
Impact of ropeginterferon alfa-2b on NLR dynamics and correlation with JAK2 V617F allele burden in the Low-PV study. **(A)** shows that Ropeg induced a rapid and sustained reduction in median NLR over 12 months compared with phlebotomy alone, both in the overall study population and, more strikingly, in patients with elevated baseline NLR (≥3.3). In contrast, NLR remained unchanged or increased slightly in patients treated with phlebotomy. **(B)** demonstrates the close relationship between inflammatory and molecular responses. Progressive reductions in NLR were accompanied by increasingly greater reductions in JAK2V617F variant allele frequency (VAF). Patients without NLR improvement showed only a modest median reduction in JAK2V617F VAF (−13%), whereas those achieving NLR reductions >50% experienced an almost 60% decrease in molecular burden. These findings demonstrate that attenuation of systemic inflammation parallels suppression of the malignant clone during interferon therapy.

Findings from PROUD-PV and CONTINUATION-PV (5) support this interpretation, showing a durable reduction in NLR over time with ropeg, particularly in patients with higher baseline inflammatory burden, whereas hydroxyurea did not produce the same effect. Greater NLR reductions were associated with deeper JAK2V617F responses, supporting a model in which ropeg first exerts anti-inflammatory and immunomodulatory effects and only later achieves more substantial clonal control. **Figure 3** illustrates the contrasting effects of ropeginterferon alfa-2b (Ropeg) and phlebotomy on NLR dynamics and their relationship with molecular response.

Unlike hydroxyurea, which largely controls hematologic parameters without substantially modifying NLR, ropeg simultaneously reduces clonal burden and inflammatory activity, supporting the concept that combined JAK2V617F-NLR trajectories provide a biologically meaningful measure of disease modification.

A similar inflammation-clone relationship has been reported with ruxolitinib. In a long-term study of hydroxyurea-resistant or -intolerant PV patients treated for a median of 8.7 years, baseline NLR correlated strongly with JAK2V617F allele burden, and patients with high baseline NLR had markedly higher variant allele frequency than those with low NLR (20). During treatment, only the high-NLR group showed a sustained decline in both NLR and *JAK2V617F* burden, whereas low-NLR patients showed little longitudinal change. These findings suggest that NLR may serve as a simple surrogate marker of biologic disease activity and support the broader relevance of the inflammation-clone axis across therapeutic classes. At the same time, they do not establish whether ruxolitinib directly modifies the malignant stem cell compartment or whether the observed molecular changes are secondary to suppression of inflammatory signaling.

This distinction remains important, especially because preclinical data indicate that ruxolitinib may act predominantly through inhibition of cytokine production in both malignant and non-malignant cells. Experimental studies by Gorantla et al. support this view, showing that the therapeutic effects of JAK inhibition depend heavily on dampening the inflammatory microenvironment rather than selectively eradicating the clone (21).

Clinical data from MAJIC-PV are consistent with this framework (22). In hydroxyurea-resistant or -intolerant PV, ruxolitinib improved complete response rates, duration of response, and event-free survival compared with best available therapy, and molecular responses were more frequent and clinically favorable. However, the absence of NLR and other inflammatory biomarker data limits mechanistic interpretation. This is particularly relevant because the meaning of *JAK2V617F* reduction is complicated by co-mutations such as *TET2* and especially *ASXL1*, which may independently amplify inflammation through NF-kB, interferon, and TNF-alpha pathways. This may help explain why *ASXL1*-mutated patients have poorer outcomes on ruxolitinib despite effective JAK-STAT suppression. Whether ropeg can overcome this additional mutation-specific inflammatory program remains uncertain, and early observations suggest that adverse mutations may still predispose to vascular events, weaker responses, and clonal evolution during interferon therapy.

## 5 Mutation-specific inflammatory programs determine the clinical utility of NLR

Signaling, leukocyte-derived indices cannot adequately reflect the underlying inflammatory burden. In this setting, biomarkers more directly linked to inflammasome activation—including circulating IL-1β, IL-6, high-sensitivity C-reactive protein (hsCRP**),** and emerging proteomic signatures—are likely to provide a more accurate assessment of cardiovascular risk and therapeutic response.

These observations also have important implications for the future clinical management of CHIP. A recent comprehensive review of CHIP-associated cardiovascular disease (23) emphasized that the field is evolving from considering CHIP merely as a biomarker of biological aging toward recognizing it as a causal and potentially modifiable cardiovascular risk factor. The review highlighted several priorities for clinical translation, including identifying patients most likely to benefit from targeted interventions and integrating CHIP into routine cardiovascular practice.

Together, these observations support the emergence of precision cardio-hematology, in which therapeutic decisions and biomarker selection are tailored according to the underlying driver mutation. However, identifying the optimal biomarker for monitoring treatment response remains an important unmet need and one of the major challenges in translating CHIP biology into routine cardiovascular practice.

## 6 Mutation-specific inflammatory programs determine biomarker selection

The clinical studies discussed above establish NLR as a robust prognostic and pharmacodynamic biomarker in JAK2-mutated myeloproliferative neoplasms. More broadly, they illustrate a fundamental principle of precision medicine: the clinical utility of an inflammatory biomarker depends on the dominant inflammatory pathway activated by the underlying driver mutation.

This concept was elegantly articulated by Adamstein and Ridker (6), who proposed that the neutrophil-to-lymphocyte ratio should not be regarded as a nonspecific marker of systemic inflammation but rather as a mechanistically informative biomarker reflecting the balance between innate and adaptive immunity. Accordingly, NLR is expected to perform best in diseases characterized by neutrophil-driven inflammation and to serve not only as a prognostic marker but also as a pharmacodynamic indicator of biological response to therapy.

The strongest clinical validation of this paradigm emerged from the exploratory analyses of the CANTOS trial (23). In patients with previous myocardial infarction, selective IL-1β inhibition with canakinumab reduced major adverse cardiovascular events by approximately 60% among carriers of TET2-mutated CHIP, whereas no significant benefit was observed in individuals with DNMT3A-mutated CHIP or without CHIP. These findings provided the first evidence that distinct CHIP mutations activate different inflammatory programs and that therapeutic benefit depends on targeting the dominant cytokine pathway. Subsequent mechanistic studies demonstrated that TET2 deficiency promotes chronic activation of the NLRP3 inflammasome, resulting in macrophage production of IL-1β, IL-6 and TNF-α without substantial neutrophilia.

This mutation-specific biology explains why NLR performs differently across clonal hematopoietic disorders. In JAK2-mutated MPNs, where constitutive JAK-STAT activation drives neutrophilic myeloproliferation, NLR closely reflects disease activity and therapeutic response. In contrast, TET2-mutated CHIP is characterized predominantly by macrophage-mediated inflammasome activation, making biomarkers such as IL-1β, IL-6 and hsCRP biologically more informative than leukocyte-derived indices.

Importantly, the pharmacodynamic value of NLR extends beyond JAK2-mutated disease. In the RESCUE trial (8), selective IL-6 inhibition with ziltivekimab produced a dose-dependent reduction in NLR, primarily through a decline in circulating neutrophils. These findings demonstrate that NLR dynamically reflects modulation of the IL-1β–IL-6 inflammatory axis whenever neutrophil-driven inflammation is successfully suppressed. Thus, although NLR is not the optimal biomarker for characterizing the baseline inflammatory phenotype of TET2-mutated CHIP, it may still provide a practical indicator of biological response to targeted anti-inflammatory therapy.

Together, these observations provide the biological rationale for this review. Understanding the inflammatory program associated with each driver mutation is essential for selecting both the most informative biomarker and the most appropriate therapeutic target, thereby moving from a generic assessment of inflammation toward precision biomarker-guided cardio-hematology.

## 7 Conclusion

The evidence reviewed here supports a conceptual shift in the interpretation of the neutrophil-to-lymphocyte ratio. Rather than representing a universal inflammatory biomarker, NLR should be considered a mechanistically informative biomarker whose clinical utility is determined by the inflammatory program activated by the underlying driver mutation. Its greatest value is observed in JAK2-mutated clonal hematopoiesis and myeloproliferative neoplasms, where constitutive JAK-STAT signaling drives neutrophilic myeloproliferation and NLR consistently predicts thrombosis, survival, and response to therapy.

By contrast, epigenetic mutations such as TET2, DNMT3A, and ASXL1 generate a qualitatively different inflammatory phenotype dominated by macrophage activation, inflammasome signaling, and IL-1β/IL-6 production. In these settings, biomarkers directly reflecting cytokine-mediated inflammation—including IL-1β, IL-6, hsCRP, and emerging proteomic signatures—are likely to provide greater biological and clinical relevance than leukocyte-derived indices.

This mutation-specific framework provides a unified explanation for the heterogeneous performance of inflammatory biomarkers across CHIP and MPNs and supports a precision medicine approach in which driver mutation, inflammatory endotype, biomarker selection, and therapeutic target are considered together. Rather than adopting a “one biomarker fits all” strategy, future disease monitoring should be tailored to the dominant inflammatory mechanism, enabling more accurate risk stratification, pharmacodynamic assessment, and ultimately the development of truly disease-modifying therapies.

## Data Availability

The original contributions presented in the study are included in the article/supplementary material. Further inquiries can be directed to the corresponding author.
